# Fluorogenic Labeling Strategies for Biological Imaging

**DOI:** 10.3390/ijms18071473

**Published:** 2017-07-09

**Authors:** Chenge Li, Alison G. Tebo, Arnaud Gautier

**Affiliations:** 1 École Normale Supérieure, PSL Research University, UPMC Univ Paris 06, CNRS, Département de Chimie, PASTEUR, 24 rue Lhomond, 75005 Paris, France; chenge.li@ens.fr (C.L.); alison.tebo@ens.fr (A.G.T.); 2 Sorbonne Universités, UPMC Univ Paris 06, ENS, CNRS, PASTEUR, 75005 Paris, France

**Keywords:** fluorogenic probes, protein labeling, RNA labeling

## Abstract

The spatiotemporal fluorescence imaging of biological processes requires effective tools to label intracellular biomolecules in living systems. This review presents a brief overview of recent labeling strategies that permits one to make protein and RNA strongly fluorescent using synthetic fluorogenic probes. Genetically encoded tags selectively binding the exogenously applied molecules ensure high labeling selectivity, while high imaging contrast is achieved using fluorogenic chromophores that are fluorescent only when bound to their cognate tag, and are otherwise dark. Beyond avoiding the need for removal of unbound synthetic dyes, these approaches allow the development of sophisticated imaging assays, and open exciting prospects for advanced imaging, particularly for multiplexed imaging and super-resolution microscopy.

## 1. Introduction

Cells and organisms are complex machines driven by a set of dynamic biological events tightly orchestrated in space and time. Our understanding of their inner workings is intricately related to our ability to observe how their constituents (e.g. proteins, nucleic acids, lipids, glycans, and metabolites) organize and interact. Among all the imaging modalities available, light microscopy has revolutionized biological research. Microscopes that enable fluorescence imaging in live cells and animals have been indispensable in our current understanding of biological processes. Nowadays, the recent advances in optical fluorescence microscopy allow the observation of the dynamics of biomolecules in 3D at sub-second resolution and at the diffraction limit or below.

Beyond advances in optics and detectors, biological imaging has strongly benefited from the development of molecular tools to fluorescently label biomolecules. The most widely applied approach in cell biology is the use of autofluorescent proteins (AFPs) to light up proteins, organelles, cellular structures, and cells. The ability to genetically fuse AFPs to a protein of interest provides absolute labeling specificity. AFPs allow specific identification and tracking of proteins in the complex environment of a cell, or of cells in the mosaic architecture of a tissue/organism. In the last two decades, the discovery and engineering of a large collection of AFPs with new and improved photophysical/photochemical properties have facilitated the development of multicolor imaging, the design of biosensors able to report on cellular physiology, and the blossoming of new microscopy techniques such as super-resolution microscopy.

**Figure 1:**
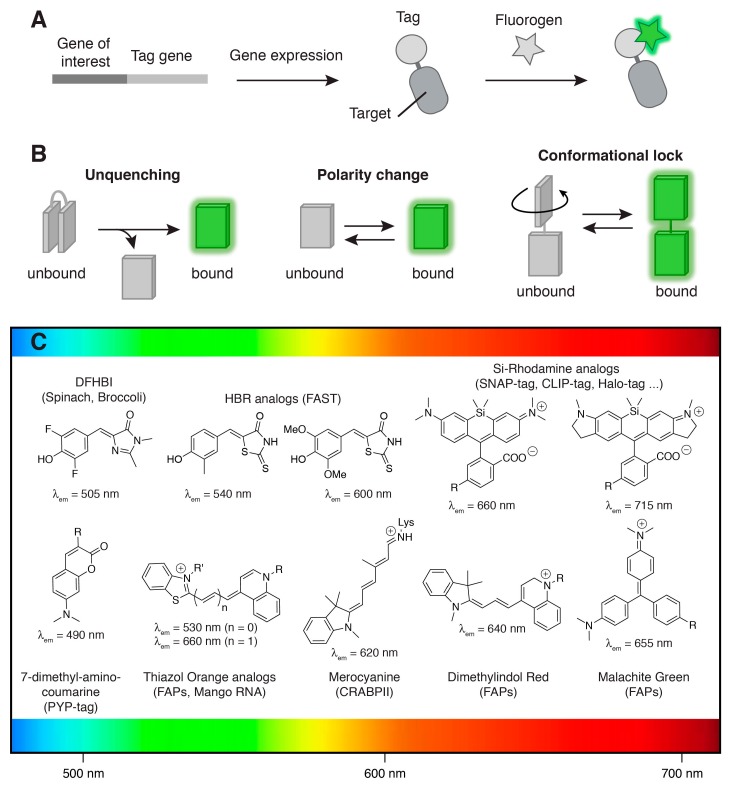
Fluorogenic labeling. (**A**) Selective fluorogenic labeling through genetic fusion to a tag (protein or RNA) able to bind a synthetic fluorogenic chromophore (so-called fluorogen) and activate its fluorescence; (**B**) Binding-induced fluorogenic response can result from various processes such as (i) unquenching of intramolecularly quenched fluorophores, (ii) fluorescence increase upon polarity change or (iii) conformational locking of molecular rotors or conjugated push-pull systems; (**C**) Main synthetic fluorogenic chromophores utilized for the development of fluorogenic labeling methods. The maximal emission wavelengths of the fluorogens bound to their cognate tag are given. Abbreviations: DFHBI = 3,5-difluoro-4-hydroxybenzylidene imidazolidinone; HBR = 4-hydroxybenzylidene rhodanine. The design of (**B**) was inspired from Reference [1].

Recently, the fluorescence toolkit has been expanded with methods for labeling biomolecules with exogenously applied small synthetic fluorescent probes. These innovative technologies offer additional labeling refinement and broaden fluorescent labeling to more diverse cellular molecules, such as RNA. Selectivity is ensured through fusion to a genetic tag that binds selectively tailored fluorescent molecules. The modular nature of such an approach enables one to tune the synthetic part by molecular engineering, in order to address biological questions with the molecular diversity offered by modern chemistry. To be usable within living systems, the genetic tag must fold and function in various cellular compartments, while the fluorescent probes must be non-toxic, membrane-permeant, and must not show unspecific interaction/reaction with cell components. A way to avoid unspecific background in cells and achieve high imaging contrast is to use fluorescent probes that display no fluorescence until labeling occurs (Figure 1A). Such probes are often called fluorogenic probes to highlight their ability to generate fluorescence upon reaction/interaction with their target. Ideal fluorogenic probes display large binding-induced changes (>100-fold) of the fluorescence intensity to allow the visualization of labelled targets over freely diffusing probes. Fluorogenic response upon binding can be achieved by changes in fluorescence quantum yield, spectral position or chromophore absorption coefficient (Figure 1B,C) [1,2,3]. In this review, we present a brief overview of recent labeling approaches that achieve high imaging contrast relying on genetically encoded protein or RNA tags that bind and activate fluorogenic synthetic molecules (so-called fluorogens).

## 2. Covalent Versus Non-Covalent Labeling

Labeling with fluorogenic probes can be covalent, relying on chemical or enzymatic reaction, or non-covalent, relying on binding equilibrium. Covalent strategies provide new experimental possibilities such as pulse-chase labeling for the study of protein synthesis, trafficking, and turn-over [4,5]. In addition, imaging contrast can be further increased due to the possibility of washing away excess probes. Non-covalent labeling strategies also offer exciting prospects. Labeling can be very fast since no covalent bond has to be created. Moreover, when the dissociation rate is sufficiently high, washing away the fluorogen can reverse labeling, switching off fluorescence. Systems displaying a high dissociation rate also have the potential of displaying increased photostability because of continuous fluorogen recycling. Finally, fine-tuning of the on rate and off rate constants can provide “blinking” systems that could be well suited for super-resolution microscopy. A potential downside of non-covalent labeling is that the probe must be present throughout the experiment, requiring careful upstream studies of their toxicity and their influence on cellular processes.

## 3. Covalent Fluorogenic Labeling

Early examples of fluorogenic probes reacting with peptidic tags are FlAsH and ReAsH, two biarsenical derivatives of fluorescein and resorufin that bind to proteins tagged with a tetracysteine motif with very high affinity and a large increase in fluorescence intensity [6]. Bis-1,2-ethanedithiol (EDT) adducts FlAsH-EDT_2_ and ReAsH-EDT_2_ are weakly fluorescent, however, when EDT is replaced with the tetracysteine tag, FlAsH and ReAsH become strongly fluorescent, lighting up proteins in live cells. The small size of the tetracysteine motif makes it one of the smallest genetic tags and is a proven advantage over AFPs [7,8,9]. However, tetracysteine labeling is limited to reducing environments, suffers from spontaneous unspecific background staining, and displays low signal-to-noise ratio when imaging low-abundant or diffusing proteins. Despite these limitations, the development and optimization [10] of the biarsenical-tetracysteine tagging system remains a landmark demonstration of the power of coupling synthetic fluorogenic probes to proteins.

Labeling selectivity was improved through the development of self-labeling tags, albeit at the expense of the tag size. The most advanced technologies nowadays are the commercially available SNAP-tag, CLIP-tag, and Halo-tag [11,12,13,14,15,16,17]. SNAP-tag is a 20 kDa protein evolved from the human DNA repair protein *O*^6^-alkylguanine-DNA alkyltransferase (AGT) [11,12,13,14,15]. SNAP-tag transfers the functionalized benzyl group of *O*^6^-benzylguanine (BG) derivatives to its active site cysteine, thus allowing irreversible covalent labeling of fusion proteins. SNAP-tag accepts a broad variety of chemical functionalities on BG, making it one of the most versatile tags currently available. CLIP-tag is an engineered variant of SNAP-tag reacting selectively with *O*^2^-benzylcytosine (BC) substrates instead of BG. SNAP-tag and CLIP-tag are fully orthogonal reactivity-wise enabling multicolor protein labeling [16]. Halo-tag, on the other hand, is a 33 kDa protein engineered from a bacterial haloalkane dehydrogenase [17]. Halo-tag was designed to covalently bind chloroalkane ligands. Early developments of fluorogenic SNAP-tag substrates relied on Förster Resonance Energy Transfer (FRET) between a fluorophore and a quencher [18]. Upon reaction with SNAP-tag, the fluorophore and the quencher are physically separated, unquenching the fluorophore and thus leading to a large fluorescence increase. This work showed that fluorogenic SNAP-tag labeling was well suited to study protein activity in real-time with high temporal resolution. Varying the chemical nature of the fluorophore and the quencher provided intramolecularly quenched substrates for multicolor labeling [19]. Note that the use of intramolecularly quenched substrates is a general strategy that has been also used with other self-labeling tags, such as trimethoprim-based chemical tags [20]. More recently, silicon-rhodamine (SiR) dyes allowed the generation of far-red fluorogenic substrates for SNAP-tag, CLIP-tag, and Halo-tag [21]. The fluorogenic response in these substrates relies on a ground state isomerization that breaks the dye conjugation. In an aqueous solution, SiR adopts mainly a closed, UV absorbing, spirolactone form, while it undergoes ring opening in less polar environments such as protein vicinity, giving a far-red fluorescent zwitterionic opened form strongly absorbing at 640–650 nm and emitting at 660–670 nm. The high cell-permeability and fluorogenicity of SiR-based substrates allowed the imaging of fusion proteins in living cells and tissues. The high photostability of SiR (and analogous azetidine containing JF646 [22]) has proven to be particularly adapted for super-resolution imaging in live cells with Stochastic Optical Reconstruction Microscopy (STORM) and Stimulated Emission Depletion (STED) microscopy. Furthermore, SiR was exploited to design fluorogenic probes for actin and tubulin imaging in living cells [23]. These probes allowed the imaging of cytoskeletal structures in living cells, such as the microtubules of the centrosome or ring-shaped actin-containing structures in axons, with sub-diffraction resolution using STED. Two-color super-resolution imaging was recently made possible thanks to the development of a SiR derivative with excitation and emission maxima at 690 and 715 nm, respectively [24]. Fluorogenic SNAP-tag substrates were also obtained using the solvatochromic membrane dye Nile Red. SNAP-tag anchoring of Nile Red allowed specific labeling of cell membrane-anchored proteins through selective activation by the proximal plasma membrane [25].

The size of AFPs and self-labeling tags is a general concern when tagging a protein. Biologists are always seeking for small genetic tags to minimize steric and functional perturbations in fusion proteins and reduce the size of the genetic material to be introduced within genomes. The last decade has seen the development of small protein tags specifically designed for fluorogenic covalent labeling. The first small tag proven to be well suited for fluorogenic protein labeling is the PYP-tag based on the 14-kDa photoactive yellow protein (PYP) from *Halorhodospira Halophila*. PYP is a monomeric blue-light photoreceptor whose sensing ability is due to the photoisomerization of its parahydroxycinnamic acid chromophore covalently attached as a thioester to Cys69. PYP and its ligands are not present in animal cells, allowing its use as a bioorthogonal genetic tag. As a labeling tag, apo-PYP was shown to react selectively with coumarin and cinnamic acid thioester derivatives through transthioesterification reactions [26,27]. The first generations of PYP fluorogenic substrates relied on quenching mechanisms based on intramolecular association between a fluorescent dye and a quencher [26,27]. Their use was hampered, however, by rather slow labeling kinetics. Rapid fluorogenic labeling of PYP-tagged proteins in live cells was achieved by using 7-dimethylaminocoumarin thioester derivatives [28]. 7-dimethylaminocoumarin derivatives are environment-sensitive fluorophores that are barely fluorescent in polar aqueous milieu, but fluoresce in the non-polar environment of a protein interior [28]. Mutagenesis of PYP combined with engineering of the electrophilicity of the thioester derivatives allowed further improvement of brightness and binding kinetics [29,30]. Recently, fluorogenic PYP-tag probes with various fluorescence colors were designed for the spatiotemporal study of proteins in living cells [31]. The use of probes of different colors that can label selectively cytoplasmic or plasma-membrane proteins allowed the understanding of the precise effect of the *N*-glycan of Glucose transporter 4 (GLUT4) on its insulin-dependent intracellular transport [31].

Apart from PYP, other small protein scaffolds have been considered as genetic tags with minimal size. Recently, the 15-kDa cellular retinoic acid binding protein II (CRABPII) was transformed into a small protein tag for fluorogenic labeling. CRABPII is a transport protein binding cellular retinoic acids. CRABPII is known to bind various synthetic retinoids and to tolerate mutations. A non-fluorescent, cell-permeant merocyanine aldehyde precursor was proven to efficiently label CRABPII mutants bearing a binding site lysine in bacteria [32]. Within minutes, formation of a protonated iminium gives a strongly red fluorescent cyanine dye, whose fluorescence is maximized by the reduced torsional freedom within the CRABPII cavity. A similar fluorogenic strategy was recently used to evolve a microbial rhodopsin into a protein displaying bright and near-infrared fluorescence [33]. Through an elegant directed evolution approach, Archaerhodopsin-3 was engineered to bind a synthetic merocyanine retinal in place of the natural retinal, and optimize the fluorescence properties of the resulting covalent complex.

## 4. Non-Covalent Fluorogenic Labeling

Parallel to the development of covalent fluorogenic protein labeling strategies, methods based on the non-covalent interaction between a protein tag and a fluorogenic dye have emerged. Non-covalent labeling provides experimentalists with novel exciting possibilities to label proteins on demand in a fully reversible fashion (*vide supra*). Most non-covalent fluorogenic labeling techniques exploit the fluorescence increase observed in some push-pull fluorogenic dyes upon immobilization. Several excited-state processes can be responsible for the environmental sensitivity of these dyes. The disruption of dye planarity through internal rotation is a source of nonradiative relaxation of the excited state through twisted intramolecular charge transfer (TICT) in polar media. Internal conversion effected by isomerization about a double bond is another source of low fluorescence. In both cases, fluorescence increase can be observed in protein cavities that are able to slow down internal rotations or isomerization, as was shown in early works with the generation of antibodies activating the fluorescence of fluorogenic molecular rotors [34] and trans-stilbenes [34,35,36].

Genetically encodable fluorogen-activating proteins (FAP) generating fluorescence through the immobilization of fluorogenic molecular rotors were evolved from single-chain antibodies [37]. FAPs binding modified thiazole orange (TO) and malachite green (MG) were first generated by screening a yeast surface-displayed library of human single-chain antibodies (scFvs) by fluorescence-activating cell sorting (FACS). Selected FAPs bind TO and MG with nanomolar affinity and increase their respectively green and red fluorescence to brightness levels encountered in AFPs [37]. These first FAPs contained internal disulfide bonds, which restricted their use to non-reducing environments such as the cell surface and secretory pathways [37]. The engineering of disulfide-free FAPs improved labeling in the cytoplasm and various other reducing subcellular compartments [38,39]. Selection of scFvs against other fluorogens successfully extended the chromatic palette of FAPs [40,41]. Of particular interest, some scFv promiscuously activate various dimethylindol red (DIR) analogs, providing access to wavelengths ranging from the blue to the near infrared [40].

Beside the far-red emitting MG-ester that shows good cell permeability and enables efficient labeling inside living cells [39], most FAP’s fluorogens are poorly cell permeant because of the presence of charges that prevent membrane crossing. This inability to cross membranes was positively used to selectively label membrane proteins without labeling the intracellular pool of proteins [42,43]. MG derivatives with optimized cell exclusion and labeling kinetics allowed the quantitative analysis of endocytosis and recycling of FAP-tagged receptors by simple add-and-read protocols [44]. This approach provides an experimental simplicity not encountered with traditional surface immunofluorescence assays, opening great prospects for high-throughput quantification of cell surface proteins using high-throughput flow cytometers [45,46,47] and plate readers [48,49]. In addition, pulse-chase labeling with two fluorogens of different colors was shown to allow for the quantification of receptor recycling through the ratiometric measurement of internalized versus non-internalized receptors upon agonist activation [48,50].

These fluorogen-based reporters were furthermore shown to open great prospects for super-resolution microscopy and single molecule tracking. Highly photostable far-red MG-based FAPs were shown to be well-suited for live cell imaging with STED nanoscopy in mammalian cells and bacteria [51,52]. The experimental control on fluorogen concentration further renders it possible to label a subset of proteins independently of their expression level, allowing the tracking of single receptors [53]. This property also enabled the random sampling of a sparse subset of emitting molecules for super-resolution imaging [54]. Stochastic sampling is normally obtained through photoswitching of (i) photoswitchable fluorescent proteins, as used in photoactivation localization microsocopy (PALM), or (ii) dyes, as used in STORM. By using the reversible binding of diffusing fluorogens present at low levels, it is theoretically possible to obtain stochastic blinking without the need for photoactivation light, because a fluorescent signal appears as a diffraction-limited spot on a target when fluorogen binds to it, while the signal turns off when the fluorogen dissociates from the target or is photobleached. Relying on such stochastic binding-based blinking, FAP-tagged proteins could be imaged with sub-diffraction resolution [54], demonstrating the great potential of non-covalent fluorogenic reporters for super-resolution imaging.

The fluorogenic toolbox was recently expanded with the development of FAST (fluorescence-activating and absorption-shifting tag). FAST is a variant of the 14 kDa PYP engineered by directed evolution to non-covalently bind hydroxybenzylidene rhodanine (HBR) derivatives and activate their fluorescence [55]. HBR and its analogs are composed of an electron-donating phenol ring conjugated to an electron-withdrawing rhodanine heterocycle. This push-pull structure deexcites non-radiatively in solution, but relaxes radiatively to the ground state within the cavity of FAST. In addition to fluorescence activation, HBR derivatives undergo an absorption red-shift of >80 nm upon binding to FAST, enabling one to distinguish bound fluorogens from free ones by the choice of the excitation wavelength, further increasing the fluorogenic response. HBR derivatives are highly cell-permeant, allowing protein labeling within a few seconds in a large number of cellular localizations and hosts [55]. FAST is the first fluorogen-based system shown to allow protein labeling in living model organisms [55]. Interestingly, the color of FAST can be experimentally tuned by using a collection of HBR analogs that give the possibility to make FAST fluoresce green-yellow, orange, or red light [56]. This spectral versatility enables one to adapt the color of FAST to the experimental spectral constraints without the need for recloning the tag, providing an experimental versatility not encountered with AFPs. Fluorogen binding in FAST is non-covalent and highly dynamic, enabling reversible labeling by fluorogen washout, making FAST a fluorescence switch that can be switched on and off at will by the addition or removal of the fluorogen within a few seconds. The ability to dynamically swap color by exchanging fluorogens emitting either green or red light provides a unique signature that was used to selectively image FAST in spectrally crowded environments. The evaluation of the degree of anticorrelation of the green and red fluorescence signals upon color swapping by two-color cross-correlation allowed the selective imaging of FAST-tagged proteins in cells expressing both green and red fluorescent proteins, although spectral discrimination was impossible in such conditions [56]. This example shows the general potential of non-covalent fluorogenic reporters for the development of new innovative imaging methods for advanced biological imaging.

Apart from protein labeling, the concept of fluorogenic labeling has great potential for observing other classes of biomolecules for which no genetically encoded fluorescent tags are available. Early studies showed that RNA aptamers could be evolved to selectively bind fluorogenic chromophores and activate their fluorescence [57,58,59,60,61,62,63], opening exciting ways for imaging RNA in living cells using aptamers as genetically encoded RNA tags [64,65,66]. The most advanced fluorogen-activating RNA aptamers for imaging RNA in living cells are from the Spinach family. Spinach [67], and its optimized version Spinach 2 [68], and Broccoli [69], are RNA aptamers that recognize derivatives of the fluorogenic 3,5-difluoro-4-hydroxybenzylidene imidazolidinone (DFHBI), an analog of the green fluorescent protein (GFP) chromophore 4-hydroxybenzylidene imidazolidinone (HBI). HBI is fluorogenic, and only fluoresces in the beta-barrel of GFP. Bare HBI is weakly fluorescent in solution, in agreement with accessible radiationless decay channels along the cis-trans photoisomerization path [70,71]. Similarly, DFHBI only fluoresces when bound and immobilized within Spinach RNA aptamers [72], which allows high contrast imaging of Spinach-tagged RNA molecules in living cells [67,68,69]. Beside RNA imaging, the Spinach technology presents great potentials for the design of fluorogenic biosensors as demonstrated by the generation of biosensors able to light up upon specific interactions with endogenous metabolites and proteins in live bacteria through the allosteric coupling of Spinach with recognition aptameric modules [73,74,75,76]. The recent development of the RNA Mango aptamer, which binds a series of thiazole orange derivatives, extended the color palette available for RNA labeling to the red edge of the visible spectrum [77].

## 5. Conclusions

Fluorogenic labeling is a general concept for imaging biomolecules with high contrast in living systems, with great potential for pushing the limit of biological imaging. Modern biology necessitates more and more advanced probes and technologies to match the increasing complexity of the questions under investigation. Fluorogenic bioorthogonal labeling methods provide an additional level of labeling sophistication as the fluorescence labeling can be controlled at will by the addition of a synthetic bioorthogonal molecule, opening great prospects for on-demand applications. Robust methods are now available for labeling proteins or RNA in various compartments in living cells and, more recently, in living model organisms, which should allow biorthogonal fluorogenic labeling technologies to become key players for the study of complex biological processes. Moreover, some of the presented technologies display unprecedented attributes—such as a small size, an oxygen-independent fluorescence, tunable spectral properties and binding-induced blinking—that make them interesting alternatives to classical autofluorescent proteins and open great prospects for advanced imaging such as super-resolution microscopies. Future developments coupling fluorogen-based reporters to sensing modules should provide powerful new technologies for measuring intracellular activities with unprecedented temporal and spatial resolutions.
